# Complete inhibition of liver acetyl-CoA carboxylase activity is required to exacerbate liver tumorigenesis in mice treated with diethylnitrosamine

**DOI:** 10.1186/s40170-024-00363-1

**Published:** 2024-11-13

**Authors:** Riya Shrestha, Calum S. Vancuylenburg, Martina Beretta, Mingyan Zhou, Divya P. Shah, Ellen M. Olzomer, Sian L. Richards, Kyle L. Hoehn, Frances L. Byrne

**Affiliations:** School of Biotechnology and Biomolecular Sciences, University of New South Wales, Sydney, NSW 2052 Australia

**Keywords:** Liver cancer, De novo lipogenesis, Acetyl-CoA carboxylase, Tumorigenesis

## Abstract

**Background:**

The metabolic pathway of *de novo* lipogenesis (DNL) is upregulated in fatty liver disease and liver cancer. Inhibitors of DNL are in development for the treatment of these disorders; however, our previous study showed that blocking DNL unexpectedly exacerbated liver tumorigenesis when liver acetyl-CoA carboxylase (ACC) 1 and 2 enzymes were deleted in mice treated with diethylnitrosamine (DEN) and fed high fat diet. Herein, we used 3 new approaches including ACC1 vs. ACC2 isotype-selective inhibition, delaying ACC inhibition until after carcinogen treatment, and feeding mice normal chow diet to better understand the impact of ACC inhibition on liver tumorigenesis.

**Methods:**

Six genotypes of female C57BL/6J mice with floxed ACC1 and/or ACC2 alleles were injected with DEN at 2 weeks of age followed by liver-specific knockout of ACC genes at 9 weeks. Mice were fed a normal chow diet and evaluated at 52 weeks for liver tumours.

**Results:**

Compared to the wildtype control group, no genotype decreased tumour multiplicity or burden; however, mice completely lacking liver ACC1 and ACC2 had > 5-fold increases in liver tumour multiplicity and burden.

**Conclusion:**

ACC inhibition exacerbated DEN-induced liver tumorigenesis only when both ACC isotypes were completely inhibited. The pro-tumour phenotype of ACC inhibition was strongly reproducible irrespective of chow or high fat feeding, and irrespective of ACC inhibition prior to or after DEN treatment. Retaining partial ACC activity at either isotype prevented tumour exacerbation in mice at risk for developing liver tumours.

**Supplementary Information:**

The online version contains supplementary material available at 10.1186/s40170-024-00363-1.

## Introduction

Liver cancer is the sixth most common cancer and third most common cause of cancer-related mortality [1]. Hepatocellular carcinoma (HCC) is the most common primary liver cancer. In recent years, the burden of HCC due to hepatitis B (HBV) and hepatitis C (HCV) infections has declined due to the development of new vaccines and anti-viral treatments [2]. Instead, obesity and related fatty liver diseases such as metabolic dysfunction-associated fatty liver disease (MAFLD) or steatohepatitis (MASH) are leading risk factors for liver cancer [2]. A hallmark of fatty liver disease is triglyceride deposits in the liver. Fats are either consumed through the diet or synthesized in the body via the process of *de novo* lipogenesis (DNL). The normal contribution of DNL to liver fat is 3–5%, but in MAFLD patients DNL contributes up to 30% [3]. Acetyl-CoA carboxylase enzymes are essential for DNL and facilitate the rate-limiting step of converting acetyl-CoA to malonyl-CoA. In mice, cytosolic ACC1 is the major isotype that drives DNL, while mitochondria-localised ACC2 is the major isotype that blocks fat oxidation. However, ACC2 can also contribute to DNL as evidenced by partial compensation in liver-specific ACC1 knockout mice [4]. Therefore, both isotypes must be inhibited to completely block DNL. ACC1 is highly upregulated in both human and mouse liver cancers [5] and there is increasing interest in inhibiting ACC activity to treat fatty liver disease and cancer [6].

In a previous study, we investigated whether liver-specific ACC1 and ACC2 double knockout using albumin-Cre transgenic mice could prevent tumorigenesis in the most common mouse model of liver cancer involving diethylnitrosamine (DEN) and Western-style high-fat diet [5]. DEN is a procarcinogen metabolized in the liver to an active carcinogen by CYP2E1 where the active molecule induces oxidative stress and DNA damage. The addition of a Western diet to the DEN model accelerates tumorigenesis compared to chow diet feeding [7]. To our surprise, mice lacking liver ACC1 and ACC2 activity developed more tumours than controls despite completely lacking the capacity for DNL [5].

In the present study, we further investigate the unexpected result where ACC deficiency increased tumorigenesis by addressing 3 gaps in knowledge. First, we investigated ACC isotype-specific effects by studying mice with 6 different ACC genotypes to determine how ACC1 vs. ACC2 expression affected tumour outcomes. Second, we investigated whether changing the diet from high fat Western diet, which supplies excess lipids available for scavenging and decreases ACC expression and activity, to low fat chow diet impacts ACC-dependent tumorigenesis. Third, we altered the timing of ACC gene knockout from before DEN treatment (albumin-Cre) to 7 weeks after DEN treatment by adeno-associated virus (AAV)-mediated gene deletion.

The results showed that mice lacking both ACC isotypes in the liver were the only group to have an altered liver tumour phenotype with markedly increased tumour multiplicity and total burden compared to wild-type control mice. These data highlight that the phenotype whereby complete inhibition of ACC activity facilitates increased tumour initiation is reproducible and is independent of diet. Furthermore, the increased tumour phenotype requires complete inhibition of both ACC1 and ACC2 activity as retaining one allele of either ACC1 or ACC2 did not increase tumorigenesis, and it is independent of whether ACC inhibition occurs before or after DEN treatment.

## Materials and methods

### Mouse breeding

All mouse experiments were conducted in accordance with the University of New South Wales (UNSW) Animal Care and Ethics Committee (Ethics approval numbers 20/43A and 23/56A). ACC1^fl/+^ / ACC2^fl/+^ male and female mice were maintained at ABR (Moss Vale, NSW, Australia) and then delivered to the Wallace Wurth animal facility at UNSW to breed and generate progeny with 6 different genotypes: ACC1^+/+^ ACC2^+/+^; ACC1^fl/fl^ /ACC2 ^+/+^; ACC1^+/+^/ACC2 ^fl/fl^; ACC1 ^fl/fl^/ACC2^fl/+^; ACC1^fl/+^/ACC2^fl/fl^ and ACC1 ^fl/fl^/ACC2 ^fl/fl^. Five cohorts of mice were generated to obtain *n* = 13–18 female mice per genotype.

### Diethylnitrosamine-induced model of liver tumorigenesis

We chose female mice for this study because tumour incidence in male mice exposed to the liver carcinogen diethylnitrosamine (DEN) is double that of females (~ 90% for males vs. ~ 45% for females) [8, 9]. Therefore, using female mice allowed us to determine whether loss of ACC isotypes increase or decrease tumour incidence. Female pups of the 6 different genotypes were injected (i.p.) with DEN (25 mg/kg) at 14 days old. The mice were monitored for 24 h post-injection, and the cages were changed after one day. The pups were weaned at three weeks old. The animals were maintained on a normal chow diet with access to normal drinking water. Mice were housed in temperature and humidity-regulated rooms on a 12-hour light/dark cycle. Genotypes were confirmed by PCR agarose gel electrophoresis. The primer sequences used were: forward primer: CTTTCCAATTCAAGGTTCTGAC and reverse primer: TACAAACGCAAGAGTCATACTGG for ACC1 floxed mice, and forward primer: TATGATGCTCCTAGTTGAGTCTTC and reverse primer: GTGAAGAAGAGGAGGCTCAGTTA for ACC2 floxed mice.

### Deletion of ACC enzymes in the livers of mice

At 9 weeks of age, all female pups were intravenously injected with AAV8-TBG-Cre virus, a recombinant adeno-associated virus 8 (AAV8) expressing Cre recombinase and thyroxine-binding globulin (TBG) as a hepatocyte-specific promoter, to delete ACC genes in hepatocytes. AAV8 viral vector expressing Cre-recombinase was purchased from Penn Vector Core (AV8.TBG.PI.Cre.rBG; titre 9.15e^13 GC/mL, Lot # CS2188L, University of Pennsylvania, Philadelphia, USA). The AAV8 vector was diluted in saline to make up 10e^10 in 100 µL and was injected via the tail vein into each mouse.

### Body composition measurements

Mouse body weights were measured weekly. At 30 weeks of age, fat mass and lean mass of mice were measured using the EchoMRI-900 (EchoMRI LLC, Houston, TX, USA) at the Biological Resource Imaging Laboratory (UNSW Sydney, Australia).

### Glucose tolerance tests (GTT) and measurements of insulin

Fed blood glucose levels were measured in mice aged 30 weeks of age at 8 AM. The animals were then fasted for 6 h but given water *ad libitum*. After 6 h of fasting, a bolus of glucose solution (2 g/kg) was injected intraperitoneally. Blood glucose levels were measured at 15, 30, 45, 60, 90, and 120 min using a blood glucometer (Accu-Check^®^ Performa II blood glucose meter, Roche, Germany). Plasma was collected from mice in the fed and fasted states to measure insulin levels using the Crystal Chem mouse insulin ELISA kit. The homeostatic model assessment for insulin resistance (HOMA-IR) was calculated using the formula:

$$\eqalign{ HOMA - IR & = (Fasted\>Glucose\>mg/dL\> \cr & \quad X\>Fasted\>Insulin\>mIU/L) \div 405 \cr}$$

### Euthanasia and tumour analysis

Mice were euthanised at 52 weeks of age. Briefly, mice were anaesthetised using 4–5% isoflurane, blood was collected via cardiac puncture and then mice were euthanised by cervical dislocation. Liver and other organs were weighed and rinsed in phosphate-buffered saline (PBS). The livers were photographed and surface tumours were counted and tumour sizes measured. The left lobe was fixed in 10% neutral-buffered formalin. Liver tumours and non-tumour tissue were separated and flash-frozen in separate tubes in liquid nitrogen and stored at -80 °C for further analysis. Tumour multiplicity was calculated as the number of surface tumours per liver per mouse. Tumour incidence was calculated as the percentage of tumour-bearing animals in a group, and tumour burden was calculated as the combined volume (mm^3^) of all tumours per mouse. The volume of a tumour was calculated using the formula $\:V=\frac{4}{3}\pi\:*radius^3$.

### Gel electrophoresis and western blotting

Frozen liver tissue was powdered on dry-ice and solubilised in modified RIPA lysis buffer (50 mM Tris-HCl, pH 8.0, 5 mM EDTA, 150 mM NaCl, 1% NP-40, 0.5% SDS, 1% SDS), and 1 mM PMSF as a protease inhibitor, and 50 mM sodium pyrophosphate, 20 mM sodium fluoride, and 50 mM sodium orthovanadate as phosphatase inhibitors. Tissues were homogenized and probe sonicated on ice, followed by centrifugation. Supernatants were collected and protein was quantified using the Pierce Bicinchoninic Acid (BCA) protein assay kit (Cat. # 23225, ThermoFisher Scientific, USA). Protein lysate was denatured in Laemmli buffer and 20 µg was loaded onto 6% gels and subjected to SDS-PAGE gel electrophoresis, followed by protein transfer to a nitrocellulose membrane. The membrane was blocked with 5% skim milk powder in TBS (20mM Tris Base and 150mM NaCl) and incubated with rabbit polyclonal ACC antibody (3662, Cell Signaling, USA) in 5% bovine serum albumin (BSA) in TBS-T (20 mM Tris Base, 150 mM NaCl and 0.1% (w/v) Tween 20) at 1:1000 dilution. Mouse anti-vinculin (V9131, Sigma-Aldrich, USA) was used as a loading control. The blot was then probed with donkey anti-rabbit IgG H&L (Alexa Fluor^®^ 680 preabsorbed, ab186692, Abcam, USA) and donkey anti-mouse IgG H&L (Alexa Fluor^®^ 790 preabsorbed, ab186699, Abcam, USA) in 3% BSA in TBS-T at 1:5000 dilution. Bands were visualized by fluorescence imaging using an Odyssey^®^ DLx Imaging System (LI-COR Bioscience, USA).

### Liver and plasma triglyceride and cholesterol content

Liver lipid was extracted using a modified version of the Folch et al. method [8]. In brief, 2:1 chloroform: methanol (v/v) was added to 25 mg powdered liver tissue. The samples were digested on a rocker for 1 h at room temperature, and 400 µL of 0.9% NaCl was added and vortexed for 30 s. The samples were centrifuged (3000 rpm, 10 min, room temperature), and the bottom phase (lipid extract) was carefully collected without disturbing the middle layer. The extract was centrifuged again, and 400 µL of the extract was transferred to a new 1.5 mL tube. The extract was speed-dried using the Turvovap evaporator (Biotage, USA). The extract was then resuspended in 0.4 mL 95% ethanol and heated to 37 °C. Ten microlitres of the extract was used to measure liver triglyceride and 20 µL was used to measure liver cholesterol level. Similarly, 5µL of plasma was used to measure triglyceride and cholesterol concentration in the plasma. Colorimetric assay was used to measure liver and plasma triglyceride (T7532, Pointe Scientific, USA) and cholesterol (TR13421, Thermo Scientific, USA).

### Immunohistochemistry

At study endpoint, the right liver lobes were fixed in 10% neutral buffered formalin at room temperature for 48 h, then stored in 70% ethanol at 4 °C. The liver tissues were embedded in paraffin blocks, and 4 μm thick sections were prepared for immunohistochemical (IHC) staining for Ki67 (marker of cell proliferation), cleaved caspase 3 (marker of cell apoptosis), and CD31 (endothelial cell marker). The antibodies used were; Ki67 antibody (ab15580, Abcam, USA), CD31 (PECAM-1) (D8V9E) XP^®^ Rabbit mAb (77699, Cell Signaling, USA) and cleaved caspase-3 (Asp175) antibody (9661, Cell Signaling, USA). IHC image analysis and quantification were performed using QuPath software (v0.5.1).

### Statistical analysis

All statistical analyses and data visualization were performed using GraphPad Prism 10.0 software unless otherwise mentioned. For tumour multiplicity and tumour burden, the Kruskal-Wallis test with multiple comparisons was performed. For tumour incidence, a Firth’s bias-reduced logistic regression model was performed followed by a post-hoc analysis in R (version 4.1.3). For all other analyses, data with normal distributions were analysed using parametric tests (One-way ANOVA) and for data without, non-parametric tests were used. Data are expressed as the mean ± standard error of the mean (SEM) or median. A p-value of < 0.05 was considered significant (* *p* < 0.05; ** *p* < 0.001; ****p < 0.0001).

## Results

**Fig. 1:**
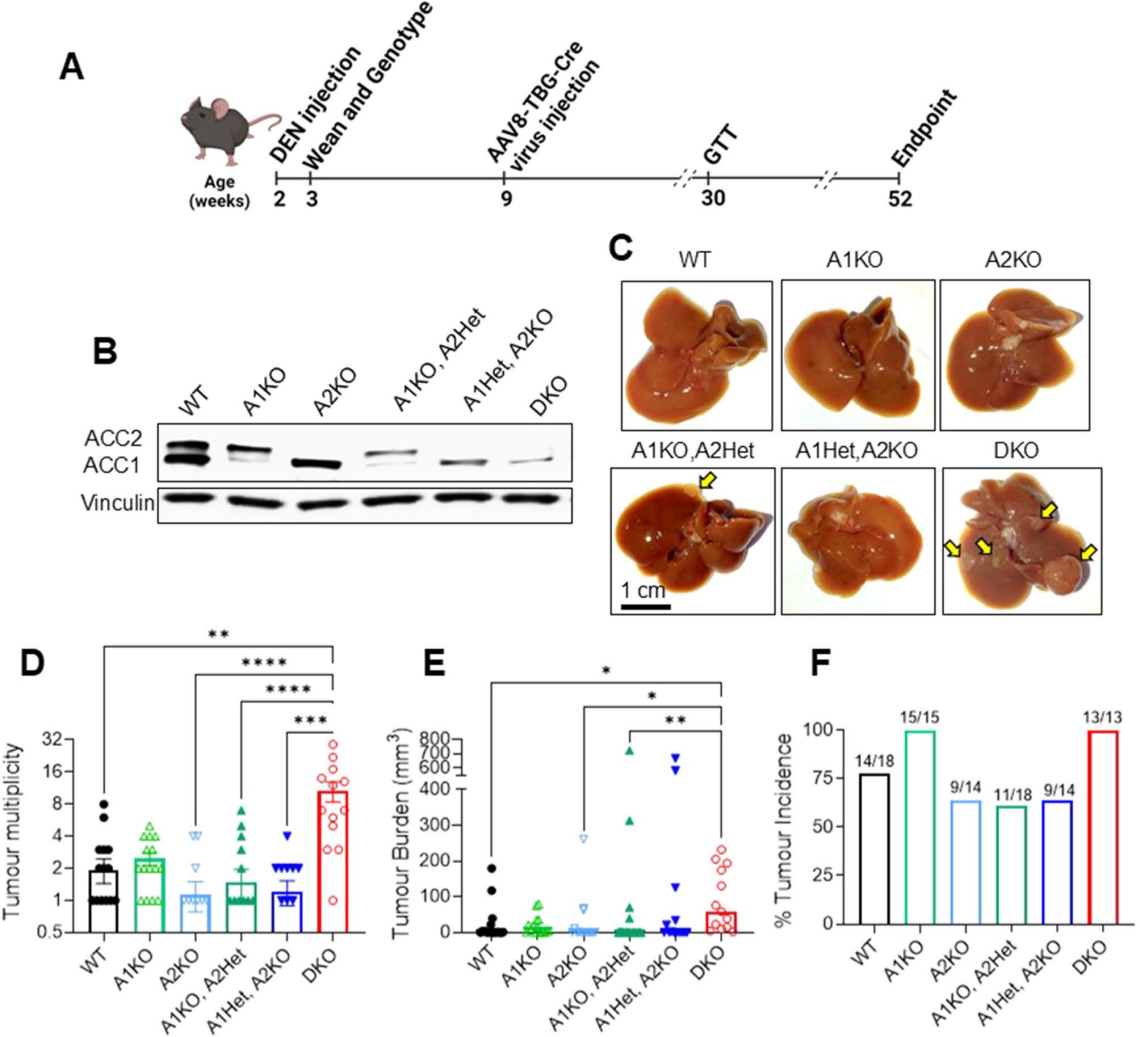
Loss of both ACC isotypes, but not individual isotypes, increases DEN-induced liver tumorigenesis in mice. (**A**) Schematic overview of the study (**B**) Western blot image of ACC1, ACC2, and vinculin (loading control) protein expression in mouse livers. (**C**) Representative image of mouse livers from each genotype. Tumours are shown with yellow arrows. (**D**) Liver tumour multiplicity, (**E**) liver tumour burden (total tumour volume in each mouse liver, mm^3^) and (**F**) tumour incidence (%) (*n* = 13–18 per group). Data are presented as means ± SEM (**D**, **G**), absolute values (**F**), or median values (**E**). Asterisks denote significant difference from DKO group (* *p* < 0.05, ** *p* < 0.01, *** *p* < 0.001, **** *p* < 0.0001)

The study timeline is illustrated in Fig. 1A. Briefly, six different genotypes of floxed ACC1 and ACC2 mice were treated with DEN at 2 weeks of age prior to AAV8-TBG-Cre mediated liver-specific ACC gene deletion at 9 weeks of age. The resulting groups included (i) Wild-type (WT) (express normal ACC1 and ACC2); (ii) ACC1 knockout (A1KO); (iii) ACC2 knockout (A2KO); (iv) ACC1 knockout with heterozygous deletion of ACC2 (A1KO A2Het); (v) ACC2 knockout with heterozygous deletion of ACC1 (A1Het A2KO); and (vi) ACC1 and ACC2 double knockout (DKO). Protein expression of ACC1 and ACC2 in mouse liver from each of the 6 genotypes are shown in Fig. 1B. Representative images of mouse livers from each genotype are shown in Fig. 1C.

Analysis of liver tumours showed the mean tumour multiplicity was 5.5-fold higher (Fig. 1D, *p* < 0.01) and median tumour burden was 40-fold higher (Fig. 1E, *p* < 0.05) in DKO mice compared to WT mice. All DKO mice had macroscopic tumours in the liver (100%, 13 out of 13), whereas 75% of WT mice had tumours present (14 out of 18) (Fig. 1F). In contrast, no other genotypes had significant differences in tumour multiplicity (Fig. 1D), tumour burden (Fig. 1E), or tumour incidence (Fig. 1F) compared to WT mice.

We found that on average most mice had 1–2 tumours in their livers, except for DKO mice which had on average 11 tumours in their livers (Table 1). We disaggregated the data to show the tumour sizes within each genotype (Table 2) and found that nearly all tumours, regardless of genotype, were between 0 and 2.5 mm. For WT mice, 88.2% were within this range and for DKO mice, 82% of tumours were within this range. Notable differences were observed in the > 2.5–5 mm range, where A1KO mice had 25.7% of their tumours in this range, whereas the other genotypes had 15.1% or less. Only the A1Het, A2KO and A1KO, A2Het mice had tumours greater than 10 mm in diameter. Overall, these data show that DKO mice had more tumours on average per mouse than other genotypes, but the size ranges were generally similar to other genotypes. This suggests that loss of ACC expression (in the DKO mice) led to increased tumour development, but not necessarily increased growth of individual tumours.

**Table 1:** Total tumour numbers per genotype and average tumours per mouse

Genotype	Total tumours	Mice (*n*)	Average tumours/mouse
WT	34	18	2
A1KO	35	15	2
A2KO	16	14	1
A1KO, A2Het	27	18	2
A1Het, A2KO	17	14	1
DKO	139	13	11

**Table 2:** Tumour sizes within each genotype

	% of tumours in range (mm diameter)				
Genotype	0-2.5	> 2.5-5	> 5-7.5	> 7.5–10	> 10
WT	88.2	2.9	5.9	2.9	0
A1KO	71.4	25.7	2.9	0	0
A2KO	81.3	12.5	6.3	0	0
A1KO, A2Het	74.1	11.1	7.4	3.7	3.7
A1Het, A2KO	70.6	11.8	0	5.9	11.8
DKO	82	15.1	0	2.9	0

To determine whether there were any differences in proliferation, apoptosis or angiogenesis in the livers between genotypes that could account for the differences in tumour development between the DKO mice and other genotypes, we evaluated Ki67 (proliferation) (Supp. Figure 1A), cleaved caspase 3 (apoptosis) (Supp. Figure 1B), and CD31 (angiogenesis) (Supp. Figure 1C) by immunohistochemistry. Results showed no significant differences Ki67 (% positive cells), cleaved caspase 3 (% positive cells) or CD31 (% positive area) staining across genotypes (Supp. Figure 1A-C). Therefore, the differences in tumour growth between the DKO mice and other genotypes were not related to these common markers associated with tumour growth and cell death.

**Fig. 2:**
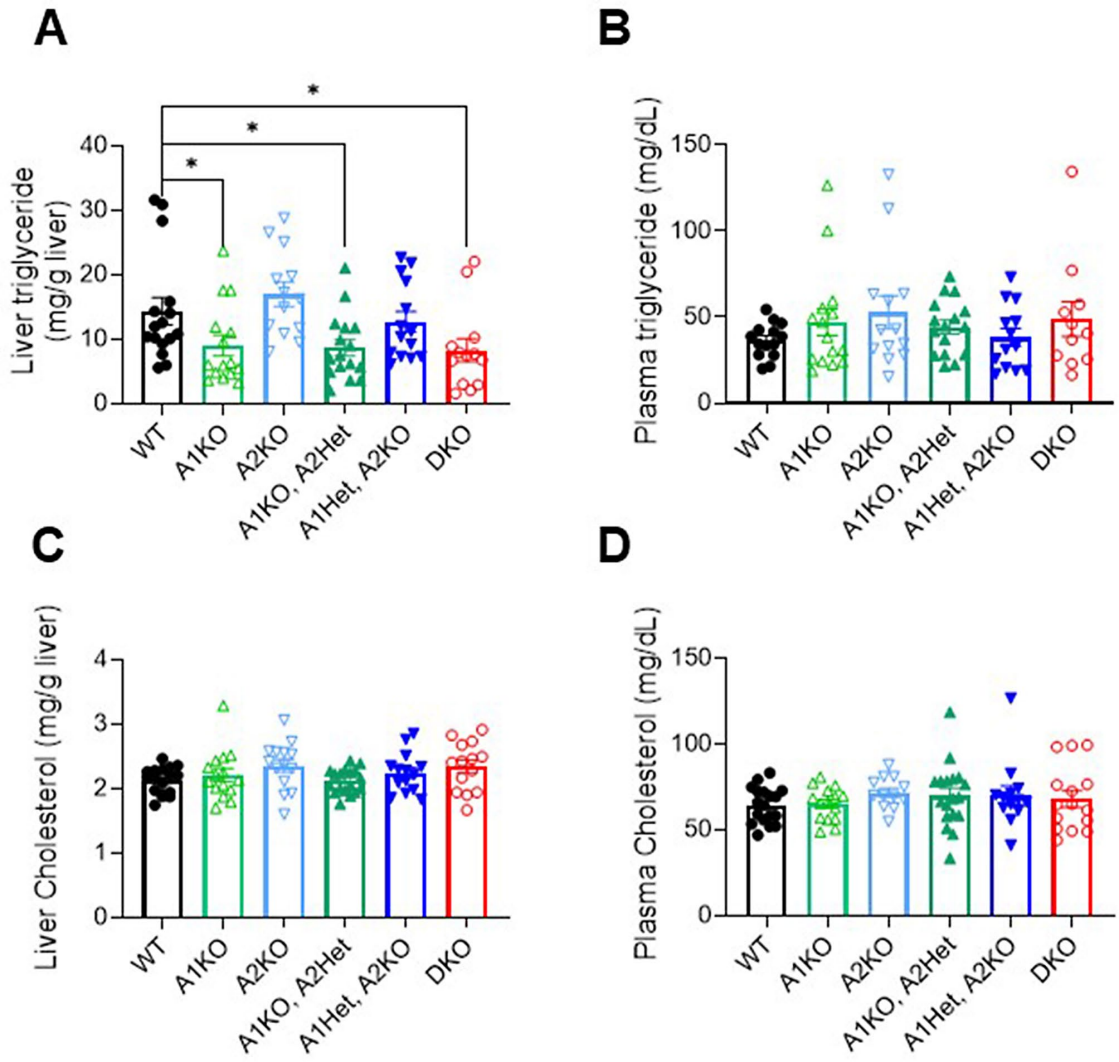
Loss of ACC isotypes in the livers of DEN-injected mice impacts liver triglycerides but has no effect on plasma triglycerides or cholesterol. (**A**) Mouse liver triglycerides, (**B**) plasma triglyceride (**C**), liver cholesterol, and (**D**) plasma cholesterol levels. Data are presented as means ± SEM. *n* = 13–18 per group

Since ACC enzymes regulate fat synthesis and oxidation, we measured triglyceride levels in mouse livers. Mice with loss of ACC1 (i.e. all 3 genotypes of A1KO, A1KO A2Het, and DKO) had ~ 40% lower liver triglycerides compared to WT mice (Fig. 2A, *p* < 0.05). Therefore, liver triglyceride levels were not independently associated with tumour multiplicity or burden. There were no significant differences between genotypes for plasma triglyceride, liver cholesterol, or plasma cholesterol (Fig. 2B-D).

**Fig. 3:**
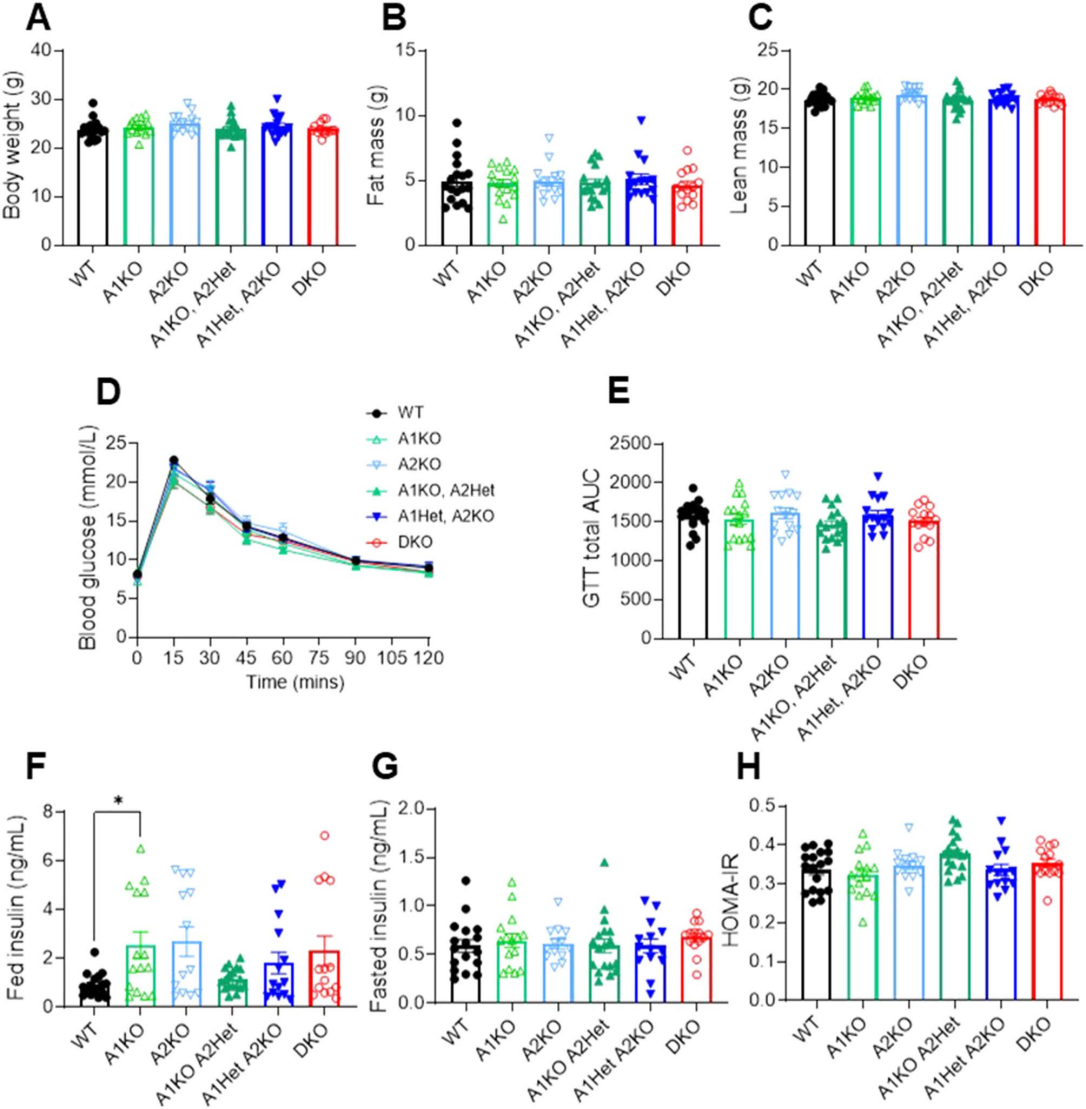
Loss of ACC isotypes in the livers of DEN-injected mice does not alter metabolic phenotypes. (**A**) Total mouse body weights, (**B**) fat mass and (**C**) lean mass at 30 weeks old. (**D**) Glucose tolerance test in 30-week-old mice and (**E**) bar graph showing the total area under the curve for the GTT. (**F**) Fed and (**G**) fasted insulin (* *p* < 0.05) were measured in 30-week-old mice, and (**H**) HOMA-IR was calculated from the fasted glucose and insulin levels to determine insulin resistance. Data are presented as means ± SEM. *n* = 13–18 per group

**Fig. 4:**
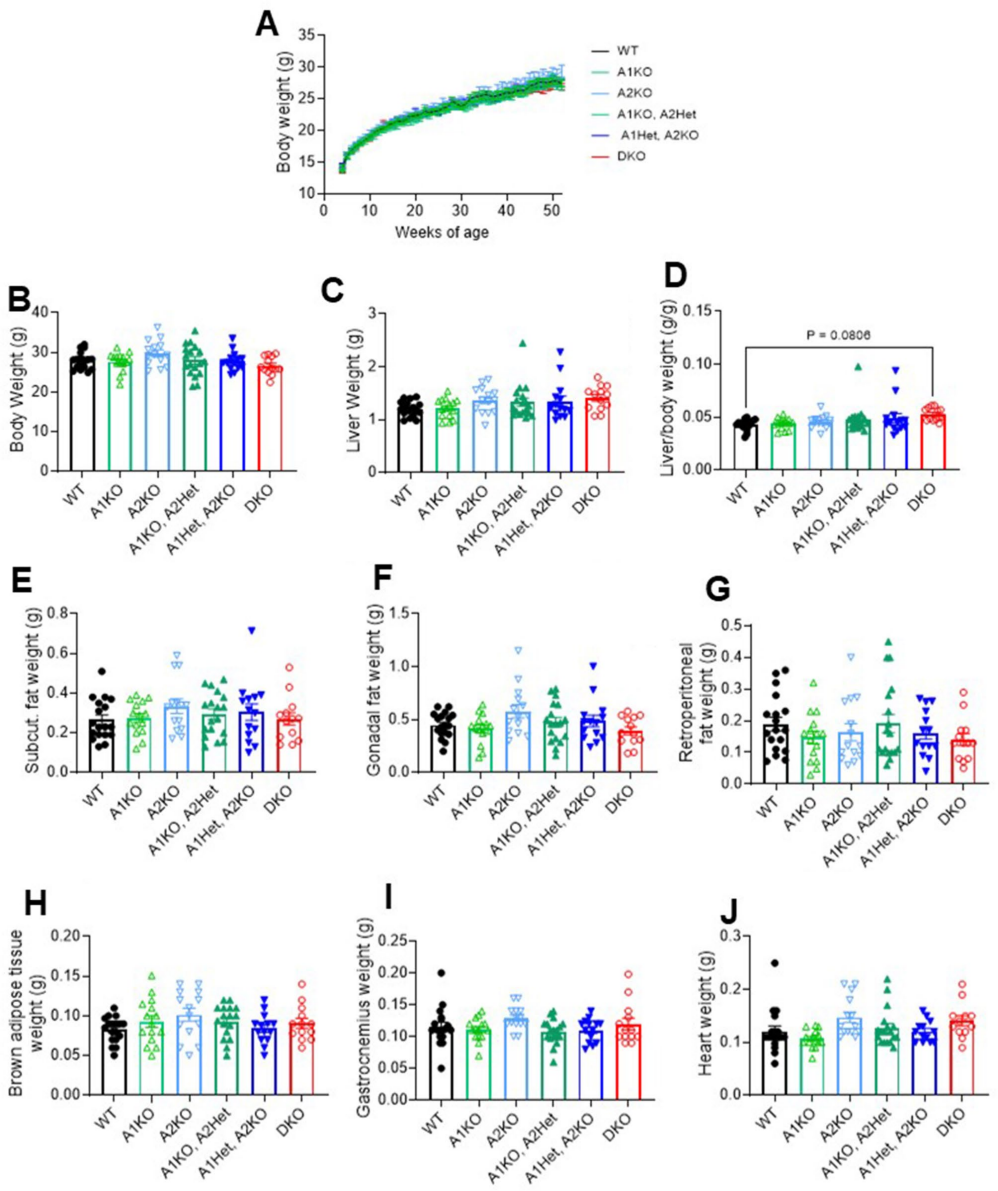
Loss of ACC isotypes in the livers of DEN-injected mice does not alter body and tissue weights. (**A**) Mouse body weights from 4 to 52 weeks of age. (**B**) Total body weights at study endpoint, (**C**) liver weights, and (**D**) liver to body weight ratio, (**E**) subcutaneous fat weights, (**F**) gonadal fat weights, (**G**) retroperitoneal fat weights, (**H**) brown adipose tissue weights, (**I**) gastrocnemius muscle weights, and (**J**) heart weights at study endpoint. *n* = 13–18 per group

Mice were assessed at 30 weeks of age (pre-tumour) for body composition (Fig. 3A-C), glucose tolerance (Fig. 3D-E), fed and fasted insulin levels (Fig. 3F-H), and HOMA-IR (Fig. 3H); however, the only phenotype observed was a 2.8-fold increase in fed insulin levels in A1KO mice vs. WT mice (Fig. 3F, *p* < 0.05). Mouse body weights were monitored throughout the study (Fig. 4A), and at the study endpoint there were no significant differences across genotypes for body weight or liver weight (Fig. 4B-C). There was a trend for a higher liver-to-body weight ratio in the DKO mice vs. WT mice (*p* = 0.08), which could be attributed to a higher tumour burden in the DKO mice (Fig. 4D). Analysis of mouse fat pads (subcutaneous, gonadal, retroperitoneal, and brown) showed no differences across genotypes (Fig. 4E-H). Similarly, mouse muscle (gastrocnemius) and heart weights were not different between genotypes (Fig. 4I-J). These results suggest that metabolic tissue weights and mouse physiology were not related to liver tumour outcomes.

## Discussion

ACC inhibitors are being trialled to treat fatty liver disease and several different types of cancer [6]. However, it currently remains unclear whether targeting ACC1, ACC2, or both isotypes represents the better intervention strategy for either fatty liver disease or liver cancer. For example, the dual ACC1/2 inhibitor MK-4074 decreased hepatic steatosis but elevated plasma triglycerides in humans [10]. The ACC1-selective inhibitor ND-630 (GS-0976, firsocostat) is 3-fold more potent towards ACC1 than ACC2 and had similar effects as MK-4074 in humans including decreased hepatic steatosis with increased plasma triglycerides, although the plasma triglyceride levels were less than MK-4074 [11]. ND-630 treatment of mice with fatty liver disease decreased liver triglyceride levels without statistically increasing plasma triglyceride [12]. Other isotype-selective ACC1-specific and ACC2-specific inhibitors are in development by Takeda, Abbott, Pfizer, and Boehringer Ingelheim but their effects in humans remain unknown.

To our knowledge, there are no published clinical data with ACC inhibitors used for the treatment of liver cancer. However, there is proof-of-concept that pharmacological ACC inhibition may be beneficial for the treatment of liver cancer as one study showed that ND-654, a derivative of ND-630 with better liver specificity and 2.7-fold selectivity for ACC1 over ACC2, reduced tumour burden by 41–55% in rats treated with DEN [13]. It is likely that ND-654 does not confer complete ACC inhibition over the course of treatment due to pharmacokinetics properties and/or its reversible inhibition of ACC enzymes. Therefore, the closest genetic intervention in our study to ND-654 would be the A1KO/A2Het mice, which models an ACC1 isotype-selective inhibitor and had a non-significant trend for 22.9% lower tumour multiplicity, 62.8% lower median tumour burden and 21.4% lower tumour incidence compared to WT mice. Although we had 18 mice in this group and the WT group, the study may still be underpowered to detect a statistical difference for partial improvements in liver tumour burden due to the high variability in the model.

Although none of the ACC genotypes statistically decreased liver tumour incidence or burden compared to WT mice, we observed that total inhibition of both ACC isotypes exacerbated liver tumour incidence (average of 11 tumours/mouse in DKO mice vs. 2 tumours/mouse in WT mice) and total tumour burden compared to WT mice. The increased tumorigenesis with total ACC inhibition aligns with our earlier study that compared only WT vs. DKO mice fed a high-fat Western diet [5]. The present study fed mice a normal chow diet to minimise lipid scavenging from the diet to make hepatocytes more dependent on lipogenesis; however, the chow-fed DKO mice still developed more tumours than WT controls. The reproducibility across both high fat and chow diets reveals the strong diet-independent effect of complete ACC inhibition to exacerbate tumorigenesis.

In the present study we changed the method of floxed ACC gene knockout to be via AAV-Cre mediated gene deletion instead of crossing floxed mice with mice expressing albumin-Cre. The albumin promotor becomes activated in neonatal mice proximal to DEN administration, thus it was unclear whether the increased tumour phenotypes in DKO mice could be due to ACC inhibition altering the process of carcinogenesis. Herein, we used a liver-selective AAV8 serotype and a liver-specific TBG promoter with delivery by tail vein injection at 9 weeks of age to ensure ACC gene knockout occurred 7 weeks after carcinogen treatment. The results showing a consistent strong tumour phenotype in DKO mice regardless of when ACC enzymes were knocked out suggests that ACC gene deletion did not affect the process of DEN-induced carcinogenesis. Thus, despite differences in timing and method of gene deletion, total loss of ACC activity enhanced DEN-induced tumorigenesis.

Liver triglyceride levels were primarily controlled by ACC1 expression where all mice lacking ACC1 (A1KO, A1KO/A2Het, and DKO) had approximately half as much triglyceride as the other genotypes expressing ACC1. However, liver triglyceride did not correlate with tumorigenesis as the A1KO and A1KO/A2Het mice had markedly less tumour burden than DKO mice despite similar liver triglyceride content.

A critical contribution of the present current study lies in the demonstration that only partial activity at one allele of either ACC1 or ACC2 can prevent the exacerbation of tumorigenesis mediated by complete ACC inhibition. No genetic intervention statistically decreased tumour incidence or burden; however, there was a trend for A2KO and A2KO-A1Het mice to have the lowest tumour multiplicity with most mice having approximately 50% less tumours than WT controls. Although not statistically significant, ACC2-selective inhibition while maintaining total or partial activity at ACC1 trended to have the best anti-cancer phenotypes.

Immunohistochemistry analysis of liver tissues revealed no significant differences in Ki67, cleaved caspase 3 and CD31 staining in the livers of mice across the 6 genotypes. This suggests that proliferation, apoptosis and angiogenesis were not different between genotypes and were not related to the increase in tumour numbers observed in the DKO mice (compared to other genotypes). However, we cannot rule out that these factors were not altered in the tumours (between genotypes) or in the livers at earlier time points when tumours were first developing. As shown in Table 1, there were on average 1–2 tumours/mouse in all genotypes (except the DKO mice) and most of these tumours were very small (0–2.5 mm range), which limited our ability to investigate differences between tumour vs. non-tumour liver tissues across the genotypes. Furthermore, another limitation of this study was that we were unable to identify a potential mechanism underlying the differences in tumour numbers between the DKO mice and the other genotypes.

In summary, this study supports the findings of our previous study, and both studies clearly show that ACC inhibition aggravates liver tumour growth in mice predisposed to developing liver cancer through DEN-induced liver damage, but only when both ACC1 and ACC2 are completely inhibited. The strongest trends for anti-cancer phenotypes were observed with complete ACC2 inhibition and at least partial activity of ACC1. Overall, these data warrant future studies with larger cohorts of A2KO mice and with ACC2-specific or ACC2-selective inhibitors in both the DEN model and other physiologically relevant pre-clinical models of HCC.

## Electronic supplementary material

Below is the link to the electronic supplementary material.

Supplementary Material 1: Immunohistochemistry analysis of Ki67, cleaved caspase 3 and CD31 staining in mouse livers

## Data Availability

No datasets were generated or analysed during the current study.
