# Supplementary material for: Complete inhibition of liver acetyl-CoA carboxylase activity is required to exacerbate liver tumorigenesis in mice treated with diethylnitrosamine
Source: Cancer Metab. 2024 Nov 13;12:34. doi: 10.1186/s40170-024-00363-1 (PMC11559202; doi:10.1186/s40170-024-00363-1)
Supplement: Supplementary file 1 — Supplementary Material 1: Immunohistochemistry analysis of Ki67, cleaved caspase 3 and CD31 staining in mouse livers [file 40170_2024_363_MOESM1_ESM.pdf]

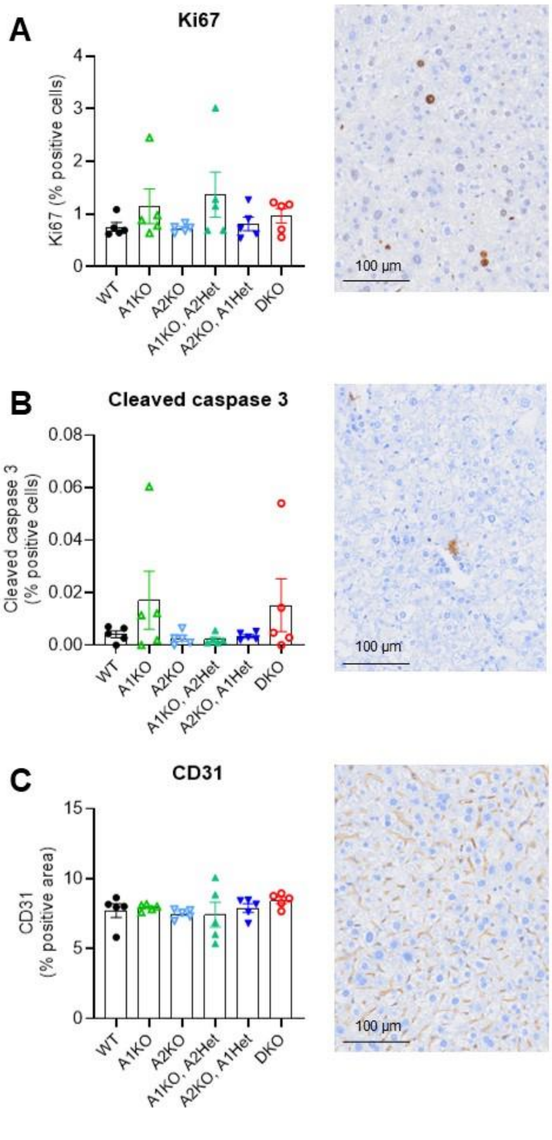


**Supplemental Figure 1**: **Immunohistochemistry analysis of Ki67, cleaved caspase 3 and CD31 staining in mouse livers.**
A) Ki67 staining in normal liver tissues from all genotypes. B) Cleaved caspase 3 staining in normal liver tissues from all genotypes. C) CD31 staining of normal liver tissues from all genotypes. Representative images for Ki67, cleaved caspase 3 and CD31 staining are shown next to the graphs. Brown staining shows the cells/areas positive for the respective marker. *n* = 5 for all genotypes.
